# The Enforcement of Sanitary Law

**Published:** 1900-05-19

**Authors:** 


### The Hospital

A Journal of

The Medical Sciences and Hospital Administration.

Vol. XXVIII.—No. 712.] Edited by Sir Henry Burdett, K.C.B., and Solomon C. Smith, M.D., M.R.C.P. [May 19, 1900.

### The Enforcement of Sanitary Law.

The discussion in the House of Commons last Thursday upon the housing of the working classes served a good purpose in that, while, on the one hand, it brought out with great force the evils of overcrowding, and the frightful extent to which it now exists in London, it also elicited from the head of the Local Government Board a very complete statement of the powers which are already in the hands of the local authorities for the abatement of the evil—powers which are to a large extent unused. It seems quite obvious that the new laws which are proposed, even if supplemented by the amendments which have been suggested, must fail of their purpose unless the sanitary laws which already exist are put into force; and we cannot but feel that Mr. Chaplin, in sketching out for the information of Parliament the amount of control over insanitary property which the local authorities already possess, passed by implication a very severe censure upon these authorities for permitting the development in their midst of those evils which were so graphically described by Mr. Steadman.

At first sight it may seem a very simple solution of the problem of overcrowding to permit a local authority to go outside its district in search of land on which to build. But it must not be forgotten that wherever it goes it will be poaching on an area belonging to someone else, and to that extent interfering with the power of some other authority to lay out its own district to the best advantage. We cannot but think that both the Government Bill and the amendments which have been proposed are founded upon a distinct misconception of the duties of a sanitary authority. Harsh as it may seem to say so, we believe that its duty is to its district rather than to the people who may happen at the time to be dwelling within it, and that just as the authority can have no possible control as to where any particular people shall take up their abode, so it has no duty to provide accommodation for any particular people who may desire at any time to dwell within its area, except so far as such an obligation is imposed upon it by the re-housing clauses of certain Acts of Parliament. The duty that really is imposed upon a sanitary authority is to ensure that such dwellings as exist, and as shall be erected within its bounds, shall be of a satisfactory character, and, unfortunately, this is just the one which, under the false idea that its first obligation is to provide a home for all who choose to dwell within their districts, the vestries are constantly neglecting. Under shelter of the plaintive query, Where are the poor people to go? the sanitary authorities of too many districts not only refuse to close premises which are clearly unfit for habitation, and in other ways to enforce the sanitary laws, but actually permit the very evils complained of to grow up under their noses. All those who have any knowledge of the working classes agree that the desire to live near their work is far greater and more urgent than any longing they may have for sanitary surroundings, and that however beautiful the cottages which it is proposed to put up in suburban districts, and however cheap the tramways by which they are to be reached, the anxiety to live near their work will always tend to cause overcrowding, and all that that connotes—especially willingness to live in and amid insanitary surroundings—in the central districts, unless such matters are very strictly looked after by those legally appointed for the purpose. Wherever rents are high and much money can be saved by submitting to overcrowding, the standard of sanitation will be certainly low unless the authorities put down a very firm foot upon all breaches of the sanitary law. If they do this, and if the County Council will ensure that the road ways which it controls are utilised to the full for their proper purposes by the introduction of every possible mechanical means of locomotion, then the question of housing will settle itself on economic lines.

Wages always will be, and always ought to be, higher in the central than in the outer circle, and so ought rents. Unfortunately central wages have not risen in the same proportion as central rents, and this is to a very great degree the fault of the sanitary authorities in not enforcing those sanitary laws which, as Mr. Chaplin showed, are now at their disposal. It is admitted on all hands that the wages now paid are not sufficient to enable the workman to provide the rent demanded for decent accommodation. Why is this? Why are not wages in the highly-rented districts higher still? Because these wages are accepted by so many people who are not keen about the decencies of life as to render it impossible for the better class of working people to raise them any higher. Surely this is largely due to the default of the sanitary authorities in permitting such conditions to go on. If, as Mr. Steadman stated, there are "no fewer than 400,000 human beings in the county of London to-day living in one-room tenements, 30,000 living six in one room, and 3,000 eight in one room," we come at once to the question, How are people who wish to live decently to obtain the wages necessary for the purpose, when they are competed with by those who, by permission of the sanitary authorities, pig together at a cheaper rate?

This question will not be solved by building houses in the suburbs, or by workmen's trains; but it would be solved to a very large extent by an unflinching enforcement of their powers by the sanitary authorities. Rents might rise, but at least the wages of the workman would rise in proportion, and would be fixed by what would support a man in decency, and not, as is now the case, by what will support one who is content to live in the midst of conditions which we all deplore.

